# Individual variations in motives for nicotine self-administration in male rats: evidence in support for a precision psychopharmacology

**DOI:** 10.1038/s41398-024-02774-6

**Published:** 2024-02-09

**Authors:** Vernon Garcia-Rivas, Jean-François Fiancette, Jessica Tostain, Giulia de Maio, Matias Ceau, Jean-François Wiart, Jean-Michel Gaulier, Véronique Deroche-Gamonet

**Affiliations:** 1 Univ. Bordeaux, INSERM, Magendie, U1215, F-33000 Bordeaux, France; 2 INSERM, Magendie, U1215, F-33000 Bordeaux, France; 3 CHU Lille, Unité Fonctionnelle de Toxicologie, F-59037 Lille, France; 4 Univ. Lille, ULR 4483, IMPECS – IMPact de l’Environnement Chimique sur la Santé humaine, F-59045 Lille, France; 5 Present Address: Yale University School of Medicine, Department of Psychiatry, New Haven, CT USA

**Keywords:** Neuroscience, Psychology

## Abstract

The significant heterogeneity in smoking behavior among smokers, coupled with the inconsistent efficacy of approved smoking cessation therapies, supports the presence of individual variations in the mechanisms underlying smoking. This emphasizes the need to shift from standardized to personalized smoking cessation therapies. However, informed precision medicine demands precision fundamental research. Tobacco smoking is influenced and sustained by diverse psychopharmacological interactions between nicotine and environmental stimuli. In the classical experimental rodent model for studying tobacco dependence, namely intravenous self-administration of nicotine, seeking behavior is reinforced by the combined delivery of nicotine and a discrete cue (nicotine+cue). Whether self-administration behavior is driven by the same psychopharmacological mechanisms across individual rats remains unknown and unexplored. To address this, we employed behavioral pharmacology and unbiased cluster analysis to investigate individual differences in the mechanisms supporting classical intravenous nicotine self-administration (0.04 mg/kg/infusion) in male outbred Sprague–Dawley rats. Our analysis identified two clusters: one subset of rats sought nicotine primarily for its reinforcing effects, while the second subset sought nicotine to enhance the reinforcing effects of the discrete cue. Varenicline (1 mg/kg i.p.) reduced seeking behavior in the former group, whereas it tended to increase in the latter group. Crucially, despite this fundamental qualitative difference revealed by behavioral manipulation, the two clusters exhibited quantitatively identical nicotine+cue self-administration behavior. The traditional application of rodent models to study the reinforcing and addictive effects of nicotine may mask individual variability in the underlying motivational mechanisms. Accounting for this variability could significantly enhance the predictive validity of translational research.

## Introduction

Nicotine is the principal psychoactive alkaloid responsible for the reinforcing properties of tobacco and the development of dependence [1]. Clinical and preclinical studies have consistently shown that nicotine strongly modulates responses to environmental stimuli, which can subsequently evolve into complex interactions between nicotine and these stimuli [2–11]. These interactions are deemed as a major factor in smoking cessation failure, despite 70% of smokers wanting to quit [12].

The significant heterogeneity in smoking behavior and motives for smoking urges [13], along with the inconsistent success of approved tobacco cessation therapies [14], suggest that these interactions do not contribute uniformly among all smokers and are mediated by distinct psychobiological mechanisms. Notably, even with one of the most effective approved pharmacotherapies [15, 16], Varenicline (Champix® or Chantix®), only 40% of treated patients achieve abstinence after a 12-week treatment [17–19]. Collectively, the evidence strongly supports the adoption of precision medicine and a departure from the “one-size-fits-all” approach toward personalized smoking cessation therapy [20].

Despite their value and effectiveness in clarifying translatable mechanisms of nicotine seeking [2–11], animal models of nicotine dependence exhibit restricted therapeutic predictive validity [21–24]. A possible explanation could be that most of these animal models do not investigate the potential variations in how individuals might be affected by the intricate nature of interactions between nicotine and its surrounding stimuli. This becomes evident with intravenous (i.v.) nicotine self-administration methods, which are the standard procedures used to study the reinforcing and motivational impacts of nicotine in animal research. These methods typically involve the simultaneous presentation of a cue [3–5], primarily visual, alongside nicotine delivery [25]. Our findings, along with those of others [26–30], have demonstrated that this type of visual stimulus is not as motivationally neutral as previously believed, as it can support instrumental responding, thus acting as a mild primary reinforcer whose value can be enhanced by nicotine [2–11]. Now, whether individual rats maintain instrumental responding to nicotine, the visual reinforcer, or an interaction between both, has remained unknown and largely unexplored.

In this study, we investigated qualitative disparities in the interactions between nicotine and the associated discrete cue as possible sources of individual variations in the psychopharmacological mechanisms underlying classical nicotine self-administration in rats. Through an unbiased cluster analysis that considered the behavioral outcomes during the selective omission of either nicotine or the cue, we identified two clusters of rats that exhibited distinct contributions of nicotine and the cue to instrumental responding during classical nicotine self-administration.

Subsequently, we compared these clusters in terms of their sensitivity to the reinforcing properties of nicotine, their response to the disruption of the nicotine-cue contingency, and the impact of Varenicline on instrumental responding to the cue in the absence of nicotine. The observed distinctions between the two clusters lend support to the idea that they pursue nicotine through distinct psychopharmacological mechanisms. One cluster primarily sought nicotine for its inherent reinforcing effects, whereas the second cluster sought it for its ability to enhance the reinforcing effects of the discrete cue. Notably, Varenicline reduced cue-seeking behavior in the former cluster, while tending to augment it in the latter cluster.

Our findings demonstrate that conventional rodent models, used to study the reinforcing and addictive effects of nicotine, potentially mask individual variability in the motivational and psychopharmacological mechanisms underlying nicotine seeking and consumption.

## Materials and methods

### Animals

Male Sprague–Dawley rats (Charles River, France), weighing 280–300 g (10 weeks of age) at the beginning of the experiments, were singly housed under a 12 h reverse dark/light cycle. In the animal housing room, temperature (22 ± 1 °C) and humidity (60 ± 5%) were controlled. Rats were familiarized with environmental conditions and experimental handling for 15 days before initiation of the experimental procedure. Standard chow food and water were provided *ad libitum*. All procedures involving animal experimentation and experimental protocols were evaluated by the Animal Care Committee of Bordeaux (CEEA50, N° 50120168-A), approved by the French MESRI (Ministry of Higher Education, Research and Innovation), and conducted in accordance with the guidelines of the European Union Directive 2010/63/EU regulating animal research.

### Surgery

A silastic catheter (internal diameter = 0.28 mm; external diameter = 0.61 mm; dead volume = 12 μL) was implanted in the right jugular vein under ketamine/xylazine anesthesia. The proximal end reached the right atrium through the right jugular vein, whereas the back-mount passed under the skin and protruded from the mid-scapular region. Ketamine hydrochloride (80 mg/kg) (Imalgène 1000; Rhône Mérieux, Lyon, France) and xylazine hydrochloride (16 mg/kg) (Rompun; Rhône Mérieux, Lyon, France) were mixed in sterile 0.9% physiological saline (saline) and administered intraperitoneally (i.p.) in a volume of 2 mL/kg of body weight.

### Experimental timeline

**Fig. 1:**
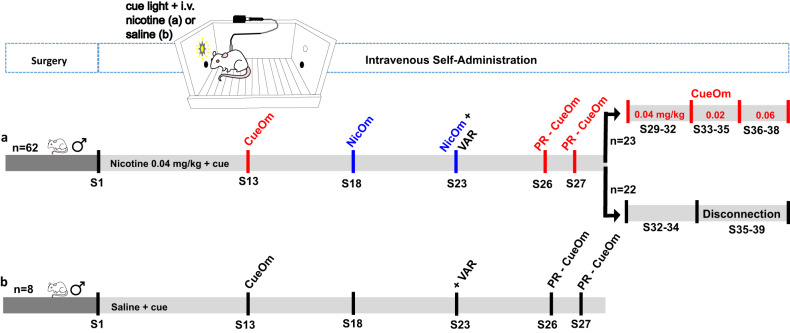
Experimental timeline. After jugular catheterization (Surgery), rats were trained in a nicotine+cue (**a**) or saline+cue intravenous self-administration protocol (**b**) with holes as manipulanda (FR3, 3 h/session). After 12 standard protocol sessions, a series of tests were performed on the whole population up to session 28: Cue omission in session 13 (CueOm), nicotine omission in session 18 (NicOm), Varenicline effect on a NicOm test (NicOm+VAR) in session 23, Progressive ratio under cue omission (PR-CueOm) in sessions 26 and 27. In sessions 29 to 38, a subgroup of representative nicotine+cue rats was tested in a dose-response for nicotine alone (CueOm), while another representative subgroup was tested from sessions 35 to 39 in a disconnection test after 7 nicotine+cue baseline sessions (28–34). The disconnection test consisted of disconnecting nicotine and cue deliveries.

After 5–7 days of post-surgical recovery, rats were trained for *intravenous nicotine + cue* (*n* = 62) or *intravenous saline + cue* (*n* = 8) self-administration, according to the experimental timeline depicted in Fig. 1. Rats were randomly assigned to the *saline+cue* and *nicotine+cue* groups, while ensuring that the two groups were balanced in terms of average body weight. The sample size for the *nicotine+cue* group was chosen, considering that the goal of the study was to conduct clustering analysis and subsequently run additional tests on independent subgroups of the identified clusters. The sample size of the *saline+cue* group, used as control for the procedure was chosen based on previous experiments from the research group [28] and was in accordance with those estimated by power analysis using G∗power software [31].

### Nicotine intravenous self-administration

The self-administration setup consisted of 48 self-administration chambers made of plexiglas and metal (Imetronic, France). Each chamber (40 cm long × 30 cm width × 36 cm high) was located in an opaque sound-attenuating cubicle equipped with an exhaust fan to assure air renewal and mask background noise. Each chamber was equipped with: (a) two holes, located at opposite sides of the chamber at 5.5 cm from the grid floor; (b) a common white light (white LED, Seoul Semiconductor, South Korea, 5 Lux), 1.8 cm in diameter, located 8.5 cm above one hole, and commonly designed as cue light; (c) a pump driving a syringe (infusion speed: 20 μL/sec) located outside the chamber on the opaque cubicle. Nose-poke visits to the two holes were recorded. (-)-Nicotine-hydrogen-tartrate (Glentham, UK) was dissolved in sterile 0.9% physiological saline and pH adjusted to 7.0 for a final training dose of 0.04 mg/kg free base, which was self-administered by the rats via intravenous route in a volume of 40 µL per self-infusion. Nicotine solutions with concentrations different (0.02 mg/kg and 0.06 mg/kg free base) than the training dose were prepared afresh and used instead of the training dose where indicated.

#### Self-administration standard protocol

At the start of the session, each rat was placed inside one chamber and connected to the pump-driven syringe through its chronically implanted i.v. catheter. Rats were trained for i.v. *nicotine + cue* or i.v. *saline + cue* self-administration on daily 3-hour sessions, running 5 days a week (Monday to Friday), except for the first session, which took place on a Tuesday. Sessions began two hours after the onset of the dark phase. Nose-poke in the active hole under an FR3 schedule produced the simultaneous activation of the infusion pump (40 μL over 2 s) and the cue light located above it (over 4 sec). Nose-pokes at the inactive hole were recorded but had no scheduled consequences. Rats were placed under an FR3 schedule of reinforcement from the first session onwards. No food training was used. Rats had no limit to the number of self-infusions available. To maintain catheter patency, catheters were flushed with ~10 µL of heparinized saline (30 IU/mL) after each self-administration session, and before the self-administration sessions run on Monday. During the standard nicotine self-administration sessions, two variables were measured: (1) the total number of infusions per session and (2) the loading proportion, calculated as the percentage of infusions achieved within 60 min. Given that rats within the same population may vary in the total amount of infusions they consume per session, we opted to normalize the initial loading to the total number of infusions [Loading proportion = (Infusions at time 60 min/total infusions) × 100]. The loading proportion captured the speed at which rats load infusions at the beginning of the session. The 60-minute time threshold was selected as it corresponds to the time when the population entered in a regular level of infusions (see Fig. S1).

#### Cue omission and nicotine omission tests

After the initial training involving 12 standard protocol sessions, we conducted cue omission (*CueOm*) and nicotine omission (*NicOm*) tests. In session 13, we assessed self-administration behavior when the cue was omitted, and in session 18, we replaced nicotine with 0.9% physiological saline to evaluate self-administration behavior during nicotine omission. Standard self-administration sessions were conducted between these two tests. For each rat, the effect of cue or nicotine omission on self-administration was evaluated using two variables: (1) The Omission Global Effect (Om-GE), calculated as the percentage change in the total number of infusions caused by the omission: [CueOm- or NicOm-GE = ([total infusions in omission test–total infusions in baseline]/total infusions in baseline) × 100]. This provides quantitative information about the overall impact of the omission test on the infusions typically achieved during baseline sessions. (2) The Omission Loading Effect (Om-LE), calculated as the difference in the loading proportion produced by the omission: [CueOm- or NicOm-LE = loading proportion in omission test–loading proportion in baseline]. This provides insights into the influence of omission on the initial loading of infusions, which can reveal temporary increases in instrumental responding known as “extinction bursts” when access to the reinforcer is suddenly removed [32, 33]. Baseline values for each type of omission effect were determined based on the two standard protocol sessions preceding the test (sessions 11 and 12 for CueOm, sessions 16 and 17 for NicOm).

### Dimensional analysis and cluster identification based on nicotine and cue omission tests

#### *Z* score normalization of cue omission and nicotine omission variables

Dimensional analysis such as principal component analysis (PCA) and clustering algorithms are sensitive to the scale of the variables. Normalizing the data using z-scoring ensures that each variable contributes equally to the analysis and prevents scale-related biases. *Z* score normalization was performed for the four variables of interest (CueOm-GE, CueOM-LE, NicOm-GE, NicOm-LE). The *z* score formula for a given individual and a given variable is the following: zi = (xi-µ)/*σ*, where xi is the value of the data point of the individual for the given variable, *μ* and *σ* are respectively the mean and the standard deviation of all dataset (*n* = 62 rats) for this variable. The *z* score normalization transforms the data in such a way that each variable has a mean of 0 and a standard deviation of 1.

#### Principal component analysis (PCA)

Using Bartlett’s test of sphericity and Kaiser-Meyer-Olkin (KMO) measure of sampling adequacy, we first assessed that our set of variables justified the use of multivariate analysis methods, such as PCA, which we performed to analyze the underlying dimensionality of the normalized *CueOm* and *NicOm* variables in our *i.v. nicotine+cue* group dataset (*n* = 62).

#### Ascending hierarchical clustering (AHC)

We investigated the grouping patterns based on the scores of the four normalized variables of interest (CueOm-GE-zscore, NicOm-GE-zscore, CueOm-LE-zscore, and NicOm-LE-zscore) using AHC. AHC does not require specifying the number of clusters beforehand and is generally less sensitive to outliers compared to other clustering algorithms like K-means. Secondly, we analyzed how the clusters identified by AHC relate to the components identified through PCA.

### Behavioral characterization of the identified clusters

The two AHC-identified clusters A and B underwent additional tests to assess their sensitivity to the primary reinforcing effects of nicotine, the reliance of instrumental responding on the contingency between nicotine and cue (disconnection test), and the impact of Varenicline on their seeking behavior.

These behavioral tests were conducted in two stages. During the first stage (session 19 to session 28), rats underwent the following tests:

***Varenicline effect on self-administration behavior supported by the cue***, conducted during session 23. Varenicline is a smoking cessation aid used to support abstinence and prevent cravings and relapse. We evaluated how Varenicline affected instrumental responding driven by the cue in the absence of nicotine (NicOm). Rats (*n* = 29 Cluster A, *n* = 26 Cluster B) participated in a NicOm session after receiving an intraperitoneal (i.p.) injection of Varenicline (1 mg/kg) 30 min before the session began. 7,8,9,10-Tetrahydro-6,10-methano-6H-pyrazino[2,3-h] [3]benzazepine tartrate (Varenicline, Tocris, UK) was dissolved in sterile 0.9% physiological saline for a final dose of 1 mg/kg free base, and administered i.p. in a volume of 2.5 mL/kg. To habituate them to i.p. injections, rats were handled and received dummy i.p. injections 30 mins before the session during the two days immediately preceding the Varenicline session.

***Motivation for nicotine self-administration through a progressive ratio***, conducted during sessions 26 and 27: We assessed the strength of the primary reinforcing effects of nicotine by measuring the rats’ motivation to self-administer nicotine (*n* = 34 Cluster A, *n* = 27 Cluster B). These sessions were similar to the CueOm session, except that the response-to-nicotine infusion ratio increased after each infusion (see SI for details). The breakpoint, which represents the maximum number of responses a rat performed to obtain one infusion (the last completed ratio), was the variable of interest. In all other sessions during this stage, rats performed the standard *nicotine+cue* self-administration sessions.

During the second stage (sessions 29–38), 45 rats were selected based on catheter patency and cluster membership, and then were assigned to one of two experiments: ***Behavioral impact of altering the contingency between nicotine and cue (disconnection test) (n*** = ***22)****.* Rats participated in five consecutive sessions where nicotine and cue were no longer contingent. Instead, each was independently accessible through different operanda. Nose-pokes at the previously active hole under the FR3 schedule activated only the cue light, while nose-pokes at the previously inactive hole under the FR3 schedule activated the pump-associated syringe, resulting in the delivery of a nicotine infusion. In order to facilitate the rats’ learning that the previously inactive hole was now reinforced by nicotine infusions, nose-pokes at the active holes were not reinforced for the first 20 min of the initial disconnection session, encouraging exploration of the inactive hole. ***Sensitivity to the primary reinforcing effects of nicotine through dose-response for nicotine self-administration (n*** = ***23)****.* Rats participated in sessions that were identical to the *CueOm* session, except that the training nicotine solution (0.04 mg/kg) was replaced by a solution containing either 0.02 or 0.06 mg/kg of nicotine-free base. Rats completed at least three consecutive sessions with each new dose. Rats from the *saline+cue* group underwent the same tests as the *nicotine+cue* group until session 28.

### Quantification of plasma nicotine and metabolites

Immediately after the end of session 21, 400 µL blood was gently collected from the catheter and immediately replaced with an equivalent volume of 0.9% saline, in 12 *nicotine + cue* rats. Blood was put in heparin-containing microtubes (Sarsted 41.1393.005), mixed and placed immediately on ice. Samples were kept on ice until centrifuged (760 G, 10 min, 4 °C). Once plasma was separated, 100 µL were carefully pipetted out, placed in 500 µL Eppendorf tubes and stored at −80 °C up to quantification. Nicotine (NIC) together with its main metabolites, cotinine (COT) and 3 hydroxycotinine (OHCOT), were measured in these plasma samples using a liquid chromatography with tandem mass spectrometry detection (LC-MS/MS) method (see SI for details). In line with the literature, we utilized the ratio of the main metabolite (cotinine) to the parent drug (nicotine) as an index for metabolism. Unlike in humans, where the two primary metabolites, cotinine (COT) and hydroxycotinine (OHCOT), are found in close range, rats exhibit low levels of OHCOT compared to COT. This disparity makes the OHCOT/COT ratio (referred to as the nicotine metabolite ratio or NMR) less relevant in rats.

### Statistics

Self-administration behavior was analyzed using one-way or repeated measures ANOVA with Time [sessions or time (min) within session], Hole (active *vs* inactive), treatment (NicOm *vs* NicOm+VAR) as within-subject factor, and Cluster (Cluster A *vs* Cluster B) as between-subject factor. Significant main effects or interactions were explored by pairwise comparisons of means using the Duncan post hoc test. A linear regression was performed to test the relationships between the cotinine/nicotine ratio and the number of nicotine self-infusions. A Grubbs test was performed to statistically verify the existence of an outlier for one of the variables of the disconnection test.

ANOVAs, linear regression and Grubbs test were performed using the STATISTICA 13.3.0 (2017) data analysis software (TIBCO Software Inc, Palo Alto, CA, USA). XLSTAT was used to run PCA and AHC (Data Analysis and Statistical Solution for Microsoft Excel, Addinsoft, Paris, France, 2017). Significance was set as *p* < 0.05.

## Results

### Acquisition of self-administration

All *nicotine+cue* rats successfully acquired and stabilized their self-administration behavior during the initial 12 sessions (Fig. S2a, b, and detailed results in SI). The behavior was dependent on nicotine, as it differed significantly from the behavior observed in the *saline+cue* group (Fig. S2e, f, and detailed results in SI). Additionally, the plasma cotinine/nicotine ratio, measured in a subset of rats after a standard self-administration session, exhibited a positive correlation with the number of self-infusions (*r* = 0.92, *r*^2^ = 0.85, *p* < 0.0001) (Fig. S2c, and detailed results SI). This finding is consistent with a faster nicotine metabolism (indicated here by a higher cotinine/nicotine ratio) promoting a higher nicotine intake, as shown previously [34, 35].

### Contribution of nicotine and cue to self-administration

**Fig. 2:**
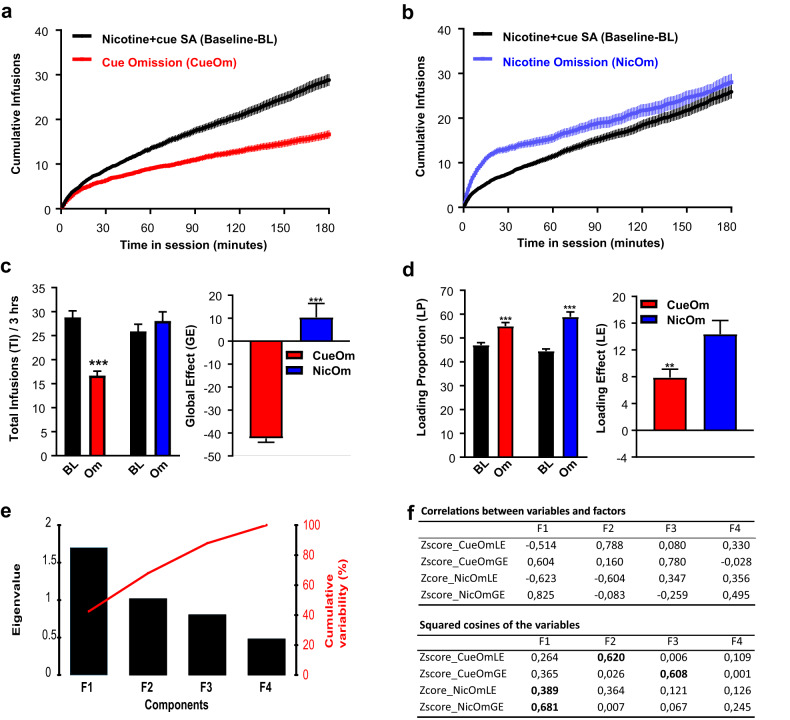
Effects of cue omission and nicotine omission on nicotine+cue self-administration behavior – Population effect. **a** Cumulative number of self-infusions over the 3-hour session. Comparison between the mean of baseline sessions 11 and 12 and session 13 (Cue Omission). **b** Cumulative number of self-infusions over the 3-hour session. Comparison between the mean of baseline sessions 16 and 17 and session 18 (Nicotine Omission). **c** Left: mean total infusions in Cue and Nicotine omissions tests (Om) and respective baseline sessions (BL); Right: The impact of omission tests on total infusions (TI), or Global effect (GE), indicated by the difference in total infusions between Omission (Om) sessions and Baseline (BL) sessions = [(TI-Om minus TI-BL)/TI-BL]x100. **d** Left: mean Loading Proportion (LP) in Cue and nicotine omissions tests (Om) and respective baseline sessions (BL); Right: The effect of omission tests on Loading Proportion (LP), or Loading Effect (LE), shown as the difference in Loading Proportion between Omission (Om) and Baseline (BL) sessions = (LP-Om minus LP-BL). LP represents the percentage of total infusions reached after 60 min. **c**, **d** ***p < 0.001 as compared to respective baseline. Data are expressed as mean ± sem. **e** Results of the Principal Components Analysis (PCA) conducted on *z* score normalized NicOm-LE, NicOm-GE, CueOm-LE, and CueOm-GE. Eigenvalues and scree plot illustrating the four components isolated by the PCA. **f** Correlations between the four CueOm and NicOm variables and the four PCA-isolated factors, along with squared cosines of the four CueOm and NicOm variables on each of the four PCA-isolated components.

Both nicotine and the nicotine-paired cue contributed to self-administration behavior. The omission of the cue (CueOm) during session 13 (Fig. 2a) and the omission of nicotine (NicOm) during session 18 (Fig. 2b) led to modifications in instrumental responding when compared to the baseline, although in distinct ways. Specifically, CueOm resulted in an ~40% reduction in total self-infusions, while NicOm tended to increase self-infusions (Fig. 2c-left), resulting in an opposed global effect (GE) for CueOm and NicOm [Global_Omission type effect, F(1,61) = 83.7, *p* < 0.00001] (Fig. 2c-right). The Loading proportion increased in both conditions (Fig. 2d-left), but with a higher loading effect (LE) in response to NicOm [Loading_Omission type effect, F(1,61) = 7.39, *p* < 0.01] (Fig. 2d-right). Large individual variations were observed in the effects of CueOm (Fig. S3a, b) and NicOm (Fig. S3c, d), including variations of opposed directions. These variations provide support for the hypothesis that individuals differ in how nicotine and the cue interact to influence their self-administration behavior.

The four variables of interest were normalized using *Z* scores before applying dimensional and clustering methods.

### Principal component analysis (PCA)—components underlying the four variables of interest

The suitability of our variables and dataset for PCA was confirmed through the Bartlett test (*p* < 0.001) and the KMO value (>0.5) (Table S1). Initially, a Pearson correlation matrix (Table S2) was employed to explore the relationships between the original variables prior to conducting the PCA itself. The Global Effect (GE) and Loading Effect (LE) variables were identified as non-redundant and capable of capturing different aspects of the omission effect. Notably, there was no proportional relationship between CueOm-GE and CueOm-LE (*r*^2^ = −0.131, ns). Likewise, NicOm-GE and NicOm-LE displayed a weak association (*r*^2^ = −0.379, *p* < 0.05). This observation was further corroborated by the PCA analysis, which revealed four components. Among the four components identified by the PCA, the inflection point on the scree plot and the eigenvalues indicated three primary components (F or Factors) that collectively explained about 88% of the total variance (F1: 42.5%, F2: 25.4%, and F3: 20%) (Fig. 2e). For both NicOm and CueOm, the two types of variables (GE and LE) loaded differently on the different PCA factors (Fig. 2f, top) and their variance was differently explained by the different factors (Fig. 2f, bottom), underscoring their differences.

Analyzing the correlations between the original variables and the components, as well as the squared cosines of the variables for each factor, unveils distinct relationships (Fig. 2f). Specifically, F1 appears to capture the response to NicOm: this primary component (F1) displays substantial loadings for the two NicOm variables (Zscore_NicOm-GE and Zscore_NicOm-LE). F2 seems to encompass the loading effect regardless of the omission type: F2 is primarily associated with Zscore_CueOm-LE, while Zscore_NicOm-LE contributes nearly equally to both F2 and F1. Lastly, F3 seems to represent the global effect of CueOm, as indicated by its stronger correlation with Zscore_CueOm-GE.

### Individual variations in the respective contribution of nicotine and the cue to self-administration: identification and characterization of two clusters (A and B) and relationships with the PCA factors

In order to explore whether distinct response patterns to CueOm and NicOm are present, we conducted Ascendant Hierarchical Clustering analysis (AHC) on the four normalized variables of interest. Our nicotine+cue dataset (*n* = 62) satisfied the requirements for minimum sample for clustering techniques (2^*d*^ where *d* is the number of dimensions) [36]. AHC identified two clusters of individuals as the optimal choice, determined by the Hartigan method (Table S3 top). Cluster A included 39 rats, whereas Cluster B encompassed 23 rats. Analysis of variance reveals significant differences between the two clusters across the four variables of interest (Table S3 bottom). However, four rats classified in Cluster A were subsequently reclassified due to receiving a negative silhouette score, which indicated misclassification.

**Fig. 3:**
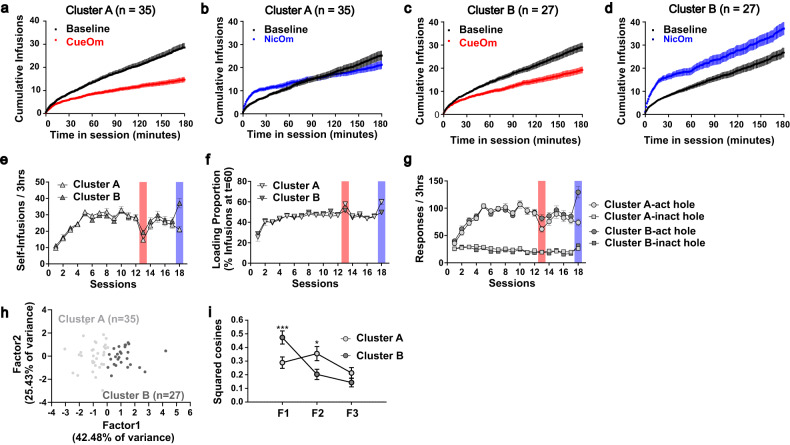
Effects of cue omission and nicotine omission on nicotine+cue self-administration behavior – Differences between the two identified clusters. **a**–**d** Cumulative number of self-infusions during baseline and omission tests, by cluster. **e**–**g** Acquisition of nicotine+cue self-administration and effects of cue and nicotine omission in the two identified clusters. Cue omission in session 12 (red bar) and nicotine omission in session 18 (blue bar) led to different behavioral alterations in the two clusters, in terms of **g** number of earned infusions per session, **f** loading proportion per session, and **g** responses in the active hole (act hole) but the inactive one (inact hole). **h** Distribution across the two primary factors resulting from the PCA analysis for Cluster A and Cluster B. **i** Disparities between Clusters A and B in terms of loading onto F1, F2, and F3 PCA factors, as measured through mean squared cosines. Data are expressed as mean ± sem. ***p < 0.0001, *p < 0.05, as compared to the other cluster.

While the two clusters differed in their response to CueOm and NicOm (Fig. 3a–d, Fig. S4, SI), they did not exhibit differences in the acquisition and maintenance of *nicotine+cue* self-administration (Fig. 3e–g).

We analyzed how the members of the two AHC-generated clusters contributed to the three main components isolated by the PCA. The members of the two clusters best segregated according to F1 coordinates, a component that translates the response to NicOm, as mentioned above (Fig. 3h). To gain further insight into the qualitative differences between the two clusters, the average cos2 of observations for each main factor was compared between clusters (Fig. 3i). The two clusters exhibited differences in their fit with the three main factors [Cluster x Factor, F(2120) = 5.58, *p* < 0.005], with Cluster B fitting better than Cluster A with F1, representing the response to NicOm, and Cluster A fitting better than Cluster B with F2, representing the loading effect regardless of the omission type.

Altogether, the qualitative and quantitative differential effects of CueOm and NicOm observed in the two clusters (Fig. 3) suggest that their self-administration behavior is supported by different interactions between nicotine and the cue. In Cluster A, both nicotine and the cue contribute to, and are necessary, to support the behavior. In Cluster B, the cue alone is capable of supporting self-administration behavior. While an initial “extinction burst” is evident, the time course of infusions eventually follows a pattern similar to the baseline (Fig. 3d). Although nicotine alone (CueOm) in Cluster B exhibits an extinction-like profile (increased loading proportion and decreased maximal infusions) (Fig. 3c), this effect is less pronounced compared to Cluster A (Fig. 3a).

### Psychopharmacological features of clusters A and B

#### Response to the reinforcing effects of nicotine: progressive ratio and dose-response curve for nicotine

To evaluate the primary reinforcing effects of nicotine, we conducted progressive ratio (PR) and FR3 dose-response tests in *CueOm* conditions.

**Fig. 4:**
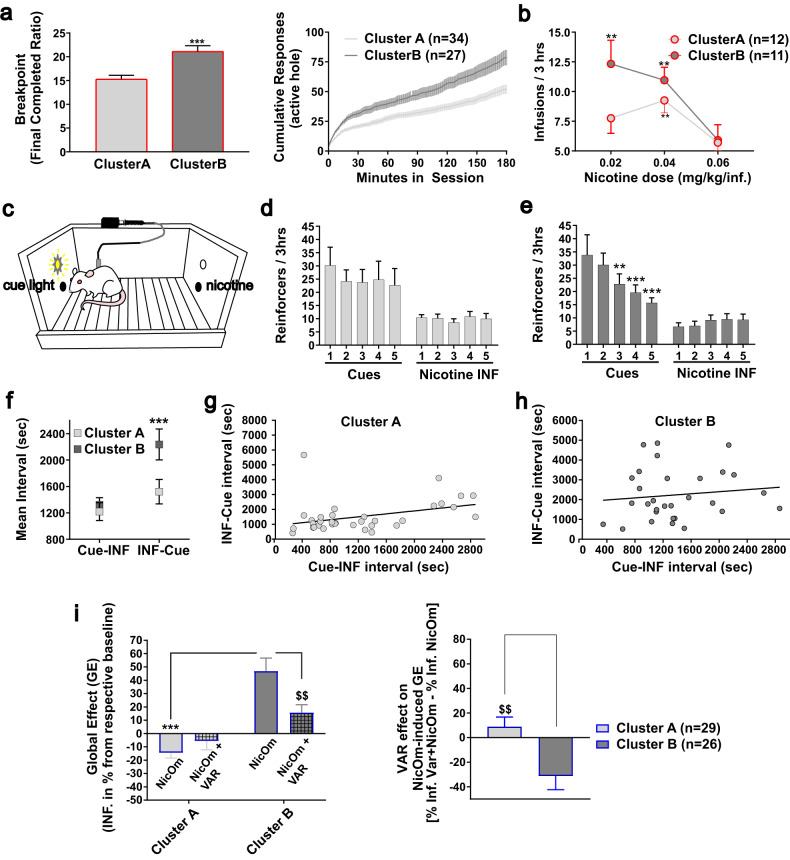
Psychopharmacological characterization of the identified clusters. **a**–left Mean breakpoint over the two progressive ratio sessions (S26 and S27) under Cue Omission (CueOm). ***p < 0.001. **a**–right Mean cumulative responses in the active hole during the progressive ratio sessions. **b** Dose-response for nicotine self-administration under CueOm. **p < 0.01 as compared to the 0.06 mg/kg dose. **c** Design of disconnection test. **d** Mean cues and infusions earned per session during the disconnection test in Cluster A. **e** Mean cues and infusions earned per session during the disconnection test in Cluster B. ***p < 0.001, **p < 0.01 compared to the first session. **f** Mean interval between cues and next infusion and between infusions and next cue over sessions 2 to 4. **g** Correlations between the two types of interval for Cluster A (*r* = 0.36, *r*^2^ = 0.13, *p* < 0.05, reaching *r* = 0.68, *r*^2^ = 0.47, *p* < 0.0001 when discarding the outlier value). **h** Correlations between the two types of interval for Cluster B. **i**–left Global effect of nicotine omission without (NicOm) or with Varenicline pre-treatment (NicOm+VAR). ***p < 0.001, ^$$^*p* < 0.01. **i**–right Compiled VAR effect on NicOm. ^$$^*p* < 0.01. **a**–**f**, **i** Data are expressed as mean ± sem.

Rats from Cluster B exhibited a higher breakpoint for nicotine self-administration (Fig. 4a-left) [Cluster, F(1,59) = 16.34, *p* < 0.0005] and sustained responding throughout the PR session (Fig. 4a-right). Data were averaged over the two sessions of PR as the difference between clusters was similar in the two sessions [Cluster x Session, F(1,59) = 0.37, *p* = 0.54, not shown].

Additionally, self-administration behavior, as measured through the mean number of injections per session, was dose-dependent [Dose effect, F(2,42) = 13.72, *p* < 0.0001]. The dose-relationship was different in the two clusters [Cluster, F(1,21) = 1.49, *p* = 0.23; Cluster x Dose, F(2,42) = 3.12, *p* < 0.05]. Cluster B maintained self-administration for the lower nicotine dose (Fig. 4b). These findings support the results from the CueOm test (Fig. 3c vs Fig. 3a), the loading of Cluster B rats on PCA factor F1 (Fig. 3h) and their highest mean squared cosine value for F1 as well (Fig. 3i), indicating that rats in Cluster B appear more sensitive to the reinforcing effects of nicotine.

#### Effect of altering the contingency between nicotine and cue (disconnection test)

To examine the nature of the interaction between nicotine and cue during self-administration, we conducted a disconnection test. In this test, the cue and nicotine delivery were dissociated and delivered through the active hole and previously inactive hole, respectively (Fig. 4c). Rats learned the new rule during the first 20 min of the first session when only the nicotine hole was active and delivered infusions, while visits to the cue hole had no scheduled consequence. During these initial 20 min, there were no significant differences between the two clusters in terms of total responding [Cluster effect, F(1,22) = 0.004, *p* = 0.95] and the time course of responding [Cluster x Time, F(1,22) = 0.32, *p* = 0.58] (Fig. S5). Notably, both clusters reached the same level of responding for the hole delivering nicotine. For this first session, there were no significant differences between the two clusters (Fig. S6).

Over the next five sessions, the two clusters did not differ for total nose-poking, including total responding over sessions [Cluster effect, F(1,20) = 0.09, *p* = 0.76; Cluster x Session, F(4,80) = 0.76, *p* = 0.55]. However, the distribution of the responses in the two holes progressed differently over sessions between the two clusters [Session x Hole x Cluster, F(4,80) = 2.69, *p* < 0.05] (Fig. 4d, e). Cluster B decreased responding in the hole delivering the cue light, with the opposite tendency for the hole delivering nicotine (Fig. 4e). Differently, Cluster A maintained a stable behavior in both holes from the first to the last session (Fig. 4d). This result further supports that standard *nicotine+cue* self-administration behavior in Cluster A is driven by nicotine-induced enhancement of cue reinforcing effects. Both the cue and nicotine are required for self-administration behavior to be maintained, but not necessarily in a contingent manner. In contrast, for Cluster B, when disconnected from nicotine delivery, self-administration of the cue alone progressively extinguishes over sessions, consistent with the cue exerting secondary reinforcing properties [37].

To gain further insight into the timing of seeking cues and nicotine, we calculated two mean time intervals: one between each cue and the next nicotine infusion, and the second between each nicotine infusion and the next cue. The two clusters exhibited significant differences [Cluster effect, F(1,62) = 4.63, *p* < 0.05], and this difference was primarily driven by the INF-Cue interval [Cluster x Interval, F(1,62) = 4.06, *p* < 0.05] (Fig. 4f). In Cluster A, we observed a time-balanced distribution of cues and nicotine infusions. The Cue-INF and INF-Cue intervals were similar (Fig. 4f) and correlated with each other [*r* = 0.36, *r*^2^ = 0.13, *p* < 0.05], supporting the hypothesis that nicotine and cue were spaced evenly (Fig. 4g). The correlation was increased (*r* = 0.68, *r*^2^ = 0.47, *p* < 0.0001) after the exclusion of an outlier confirmed by a Grubbs test (Grubbs test statistic = 3.84, *p* < 0.0005 for INJ-Cue interval). This profile is in accordance with the reinforcement-enhancement tracking of the circulating levels of nicotine [38, 39].

In Cluster B (Fig. 4f–h), however, the time distribution of cues and nicotine infusion was unbalanced and depended on which occurred first. The mean INF-Cue interval was significantly longer than the Cue-INF interval and they were uncorrelated, suggesting that nicotine was more reinforcing than the cue.

#### Varenicline effect on nicotine seeking in Clusters A and B

Varenicline had a differential effect on seeking behavior in clusters A and B when nicotine was omitted [Cluster effect, F(1,53) = 38.84, *p* < 0.00001; Cluster x Treatment, F(1,53) = 8,85, *p* < 0.005] (Fig. 4i).

As mentioned earlier, during nicotine omission sessions (NicOm), Clusters A and B exhibited significant differences compared to their respective baselines. Cluster A showed a decrease in drug-seeking behavior, while Cluster B showed an increase (*p* < 0.001) (Figs. 3b, d and 4i). Varenicline reduced this increase (NicOm+Var) in Cluster B, but it had no significant effect in Cluster A (Fig. 4i-left). Varenicline acted in opposite ways on the two clusters [Cluster effect, F(1,53) = 8.85, *p* < 0.005], decreasing seeking in Cluster B and tending to increase it in Cluster A (Fig. 4i-right).

## Discussion

In line with clinical studies, animal models used for studying tobacco addiction have consistently demonstrated the involvement of various psychopharmacological mechanisms in nicotine self-administration. However, it is important to note that animal models do not account for individual variations in the mechanisms underlying nicotine self-administration, which may limit their predictive validity [40].

To address this issue, we conducted a psychopharmacological profiling study in male Sprague–Dawley rats trained for intravenous nicotine self-administration. We employed an unbiased clustering method [41], and identified two distinct clusters (referred to as Clusters A and B) characterized by different psychopharmacological mechanisms driving their self-administration behavior. These distinct mechanisms were revealed by manipulating either nicotine or the cue in different test sessions. Furthermore, we examined the effects of Varenicline, a partial α4β2 nicotinic acetylcholine receptor agonist, on self-administration behavior supported by the nicotine-associated cue in these two clusters. Interestingly, Varenicline exerted opposing effects on self-administration behavior in the two clusters, highlighting the differential response to pharmacological interventions based on the underlying psychopharmacological mechanisms.

### Contribution of nicotine and cue to self-administration

Acquisition of secondary reinforcing properties by the cue requires time [42], while nicotine-induced enhancement of cue-reinforcing effects is an acute immediate effect [43–45].

Nicotine omission (i.e., cue self-administration alone) is commonly used as a test to evaluate the secondary reinforcing effects of the cue. Already after 12 sessions, we observed a slight but significant increase in behavior by nicotine omission (Fig. 2b), similar to Cohen et al. [37] after 10 sessions of *nicotine+cue* self-administration, indicative of the cue acting as a secondary reinforcer. Differently, Clemens et al. observed a significant decrease in total active responses by nicotine omission after 10 standard self-administration sessions [42], similar to Caggiula et al. [2] after 20 sessions, while total active responses were no more affected by nicotine omission after 41 standard self-administration sessions [42].

Procedural differences can explain that the cue acquires secondary reinforcing properties at different speeds. Factors, such as session duration, nicotine dose, cue duration, food restriction, housing conditions, could play a role individually or in interaction. The procedural differences between these studies that could contribute to the faster acquisition of secondary reinforcing properties by the cue, are the cue duration in Cohen et al. (20 sec, instead of 1 sec and 3 sec for Caggiula et al. [2] and Clemens et al. [42], respectively) and session duration and dose in our study (3 h sessions and 0.04 mg/kg, instead of 1 hr sessions and 0.03 mg/kg for the three other studies).

Cue omission, i.e., nicotine self-administration alone, is less commonly tested. Differently from nicotine omission, Clemens et al. [42] and Caggiula et al. [2] had opposite results regarding the ability of nicotine alone to sustain self-administration. In Clemens et al., behavior was maintained by nicotine alone at the same level as compared to standard sessions. Caggiulia et al. [2] observed an ~55% decrease in nicotine infusions in response to cue omission, close to the 40% decrease we observed in our study. This supports that the contribution of nicotine and cue to self-administration at the population level are differentially influenced by experimental conditions. It is speculative to attribute a procedural difference as the explanation for the discrepancy with Clemens et al. regarding cue omission. Still, in Clemens et al., rats were trained at FR1, whereas they were trained at FR5 and FR3, in Caggiula et al. [2] and our study, respectively, making the maintenance of self-administration in the absence of the cue more challenging. Another distinction is housing: rats were housed four per cage and food-restricted in Clemens et al. [42], which could generate competition and stress. Eventually, it cannot be discarded that in Caggiula et al. [2], the population of rats would be enriched in Cluster A-type rats, leading to a decrease in behavior by both cue and nicotine omission.

### Cluster A: the reinforcement-enhancing effect (REE) of nicotine as a primary driver of nicotine self-administration

Our findings indicate that rats belonging to Cluster A (comprising 55% of the tested rats) are primarily motivated by the ability of nicotine to enhance the reinforcement value of the cue light. The omission tests revealed that instrumental responding in these rats relied on the presence of both nicotine and the cue (Fig. 3a–c), even when they were independently accessible through different operanda **(**Fig. 4d–f).

Importantly, their behavior in the disconnection test aligns with previous studies demonstrating that the REE of nicotine can be observed without the need for prior learning associations or contingency with nicotine [4, 44–48].

The REE of nicotine, initially observed in animal models [4, 26, 46, 49], has been substantially documented in human studies [45, 48, 50–53]. However, the neurobiological mechanisms underlying the REE are still not fully understood [54]. It is considered one of the key factors contributing to the addictive properties of tobacco [11, 44]. Furthermore, it may be involved in withdrawal-induced sensory anhedonia, which strongly promotes relapse [55–58]. The REE hypothesis also supports the notion of “self-medication,” whereby individuals with socioeconomic or health conditions associated with limited opportunities for reward may be more prone to seeking nicotine as a means of alleviating their reward deficits [59–62].

### Cluster B: nicotine and the ‘classical’ nicotine-cue conditioning as a primary driver for nicotine self-administration

Rats in Cluster B (constituting 45% of the tested rats) appeared to be primarily driven by a combination of the primary reinforcing effects of nicotine and the nicotine-paired cue acting as a conditioned reinforcer, capable of driving self-administration even in the absence of nicotine [37, 44, 63, 64]. The omission tests revealed that their instrumental responding can be partially maintained by either nicotine or cue (Fig. 3c, d). Also, Cluster B rats demonstrated greater sensitivity to the reinforcing effects of nicotine alone compared to Cluster A rats, as evidenced by their performance in progressive ratio and dose-response tests (Fig. 4a, b). Additionally, disrupting the nicotine-cue contingency led to a decrease in instrumental responding for the cue in Cluster B rats (Fig. 4e), contrasting with the behavior of Cluster A rats (Fig. 4d). This finding supports the notion that the cue in Cluster B rats has acquired conditioned reinforcing properties that gradually extinguish over time [37, 65].

In humans, the environmental stimuli that become conditioned reinforcers due to their association with nicotine are major sources of craving in some individuals [66, 67], and thus contribute to relapse [68]. Some smokers who have been switched to de-nicotinized cigarettes report lower cravings to smoke [64, 69, 70], suggesting that the conditioned stimuli associated with smoking, such as rolling a cigarette [71], or the oropharyngeal sensations of smoking [72, 73], have become strong reinforcers. Similarly, some smokers report an increase in craving after observing friends smoking, or when visiting the places associated with smoking [9, 74–76].

Further studies would need to explore whether the observed psychopharmacological profiles remain the same after protracted nicotine exposure, and whether Cluster B-like rats would be more prone to cue-induced reinstatement.

### Varenicline can have different behavioral outcomes depending on the psychopharmacological profile of nicotine-seeking

Consistent with its nature as a partial agonist at the α4β2-containing nicotinic cholinergic receptors [77–79], we report that Varenicline can moderately enhance cue-reinforcing effects in rats self-administering i.v. *saline+cue* (Fig. S7), and antagonize nicotine-induced enhancement of cue reinforcing effects (Fig. 4i), consistent with a previous study done in our laboratory [28]. We also observed relevant differences in response to Varenicline between the clusters. In Cluster B rats, Varenicline strongly diminished the increased seeking behavior observed during NicOm, suggesting that the partial pharmacological agonism by Varenicline was enough to compensate for the removal of nicotine, bringing the seeking behavior closer to baseline. Varenicline slightly increased seeking behavior in Cluster A (Fig. 4i), consistent with a reinforcement-enhancement effect.

Varenicline was used here as a tool to explore interactions between nicotine and the cue in the observed clusters. The aim of our study was not to test Varenicline with a view to therapeutic use, i.e., applied chronically [80]. However, our data confirm that animal models such as nicotine self-administration could prove useful for precision pharmacology.

### Individual differences in nicotine seeking: an opportunity to improve preclinical models of nicotine reinforcement

Clinical data strongly suggest that individuals differ as regards the breadth of motives and mechanisms that determine the urge to smoke [for review (40)], warranting the emergence of research in precision medicine for tobacco addiction. A systematic exploration of individual variations in behavior or pharmacological responses could help improve the translational and predictive value of preclinical models of nicotine reinforcement. Exploration of individual variations in nicotine self-administration is at an early stage [35, 81, 82], but our study, together with few others, supports consistent individual differences in nicotine psychopharmacology [35, 82].

In recent years, nicotine metabolism has attracted interest as a phenotypic biomarker of heavy smoking [34] and therapeutic response [83]. While fast metabolizers are at risk for heavy smoking [34], slow metabolizers benefit from nicotine replacement therapies, and normal metabolizers benefit from treatments such as Varenicline [83]. Consistently, in rats, the rate of nicotine clearance predicted the threshold of nicotine reinforcement [35], and Varenicline reduced nicotine self-administration more in rats with a higher demand for nicotine [84]. Interestingly, while in our protocol nicotine demand was positively related to the cotinine/nicotine ratio (Fig. S2c), our two clusters expressed the same self-administration behavior in standard protocol sessions (Fig. 3e–g). It thus remains unlikely that the observed behavioral differences are due to individual differences in nicotine metabolism. However, the study by Grebenstein et al. [35] suggests that nicotine seeking might be controlled by pharmacokinetics factors in some individuals, and less so in others. Echoing the fast/slow metabolizer phenotypes in humans [34], these observations offer interesting perspectives for studying how individual differences in nicotine metabolism could drive variations in the primary reinforcer and reinforcer enhancer properties of nicotine.

Our results highlight qualitative individual variations in the mechanisms supporting *nicotine+cue* self-administration behavior, with nicotine playing either a major role as primary reinforcer or enhancer, depending on the individuals. Sved et al. [85] recall that ‘*These two actions of nicotine, primary reinforcer and reinforcer enhancer, undoubtedly relate to the high incidence of nicotine use disorder and they must also be taken into account when considering smoking cessation pharmacology*.’ Although there is still a lot to investigate, these mechanisms appear to involve distinct molecular and neurobiological substrates (for review [85]). Notably, individual variations have been observed in incentive salience attribution to drug-associated cues, with nicotine-associated specificities. In the so-called sign trackers rats (STs), food- or drug-(cocaine, opioid)associated discrete cues are both more attractive (elicit approach) and more wanted (are conditioned reinforcers) than in goal trackers rats (GTs), in which presentation of reward-associated cues elicits approach to the location of reward delivery. Regarding nicotine, Yager and Robinson [82] showed that STs rats want more nicotine-associated cue, but they do not approach a nicotine-cue more than GTs rats, demonstrating nicotine-specific mechanisms of salience attribution. This model offers the opportunity to study how individual variations in salience attribution to nicotine cues relate to individual variations in the psychopharmacological mechanisms supporting nicotine+cue self-administration.

In the footsteps of oncology, precision medicine in tobacco dependence [86–90] focuses on pharmacogenetics-based markers, i.e., the identification of genetic makers that predict response to smoking cessation drugs. However, smoking is a complex behavior and data support a polygenic contribution to vulnerability and to treatment efficacy [89]. Interplay between genetic factors could shape dependence vulnerability and variations in nicotine psychopharmacology (e.g., [91]). Therefore, behavioral psychopharmacology-based markers could efficiently complement genetic markers, and precision psychopharmacology in animal models could help define such behavioral markers in humans.

The aim of this study was to question individual psychopharmacological profiles arising at the intersection of IV nicotine delivery and the presentation of a contingent visual stimulus, the most widely used model of nicotine self-administration. Since tobacco addiction is a complex phenomenon at the intersection of social, environmental and biological factors [92, 93], further studies would need to address whether the identified psychopharmacological profiles are differently modulated by sex, protracted nicotine use, access to alternative rewards, social interactions, and stress, all factors which are known to impact drug-seeking [22, 94, 95] and which are also sources of individual variability. Finally, and of special interest to translational approaches, whether these psychopharmacological profiles predict transitioning into addiction-like nicotine seeking, and whether approved cessation therapies, like Varenicline, are more beneficial to individuals fitting a particular profile compared to the other, remains to be explored.

In summary, the individual differences found in this study could contribute to the observed complexity in both human and animal studies. They have the potential to reshape current discussions on vulnerability to nicotine addiction and to open discussions about precision psychopharmacology from a translational perspective.

### Supplementary information

Supplementary Material

## References

[1] Benowitz NL (1992). Cigarette smoking and nicotine addiction. Med Clin North Am.

[2] Caggiula AR, Donny EC, White AR, Chaudhri N, Booth S, Gharib MA (2001). Cue dependency of nicotine self-administration and smoking. Pharmacol Biochem Behav.

[3] Caggiula A, Donny E, Chaudhri N, Perkins K, Evansmartin F, Sved A (2002). Importance of nonpharmacological factors in nicotine self-administration. Physiol Behav.

[4] Chaudhri N, Caggiula AR, Donny EC, Palmatier MI, Liu X, Sved AF (2006). Complex interactions between nicotine and nonpharmacological stimuli reveal multiple roles for nicotine in reinforcement. Psychopharmacology.

[5] Garcia-Rivas V, Deroche-Gamonet V (2019). Not all smokers appear to seek nicotine for the same reasons: implications for preclinical research in nicotine dependence. Addict Biol.

[6] Bani M, Andorn A, Heidbreder C (2014). Pharmacologically, are smokers the same as non-smokers?. Curr Opin Pharm.

[7] McClernon FJ, Froeliger B, Rose JE, Kozink RV, Addicott MA, Sweitzer MM, et al. The effects of nicotine and non-nicotine smoking factors on working memory and associated brain function. Addict Biol. 2016;21:954–61. 10.1111/adb.12253 PMC4618271 25904425

[8] Shiffman S, Dunbar MS, Scholl SM, Tindle HA (2012). Smoking motives of daily and non-daily smokers: a profile analysis. Drug Alcohol Depend.

[9] Shiffman S, Dunbar MS, Ferguson SG (2015). Stimulus control in intermittent and daily smokers. Psychol Addict Behav.

[10] Stoker AK, Markou A (2015). Neurobiological bases of cue- and nicotine-induced reinstatement of nicotine seeking: implications for the development of smoking cessation medications. Curr Top Behav Neurosci.

[11] Caggiula AR, Donny EC, Palmatier MI, Liu X, Chaudhri N, Sved AF (2009). The role of nicotine in smoking: a dual-reinforcement model. Nebr Symp Motiv.

[12] Benowitz NL (2010). Nicotine addiction. N Engl J Med.

[13] Garcia-Rivas V, Deroche-Gamonet V. Not all smokers appear to seek nicotine for the same reasons: implications for preclinical research in nicotine dependence. Addict Biol. 2018;24:317–34. 10.1111/adb.12607 29480575

[14] Schuit E, Panagiotou OA, Munafò MR, Bennett DA, Bergen AW, David SP (2017). Pharmacotherapy for smoking cessation: effects by subgroup defined by genetically informed biomarkers. Cochrane Database Syst Rev.

[15] Cahill K, Stevens S, Perera R, Lancaster T (2013). Pharmacological interventions for smoking cessation: an overview and network meta-analysis. Cochrane Database Syst Rev.

[16] Hartmann-Boyce J, Stead LF, Cahill K, Lancaster T (2014). Efficacy of interventions to combat tobacco addiction: cochrane update of 2013 reviews. Addiction.

[17] Jordan CJ, Xi Z-X (2018). Discovery and development of varenicline for smoking cessation. Expert Opin Drug Discov.

[18] Niaura R, Hays JT, Jorenby DE, Leone FT, Pappas JE, Reeves KR (2008). The efficacy and safety of varenicline for smoking cessation using a flexible dosing strategy in adult smokers: a randomized controlled trial. Curr Med Res Opin.

[19] Oncken C, Gonzales D, Nides M, Rennard S, Watsky E, Billing CB (2006). Efficacy and safety of the novel selective nicotinic acetylcholine receptor partial agonist, varenicline, for smoking cessation. Arch Intern Med.

[20] Kranzler HR, Smith RV, Schnoll R, Moustafa A, Greenstreet-Akman E (2017). Precision medicine and pharmacogenetics: what does oncology have that addiction medicine does not?. Addiction.

[21] Lerman C, LeSage MG, Perkins KA, O’Malley SS, Siegel SJ, Benowitz NL (2007). Translational research in medication development for nicotine dependence. Nat Rev Drug Discov.

[22] O’Dell LE, Khroyan TV (2009). Rodent models of nicotine reward: What do they tell us about tobacco abuse in humans?. Pharmacol Biochem Behav.

[23] Le Foll B, Pushparaj A, Pryslawsky Y, Forget B, Vemuri K, Makriyannis A (2014). Translational strategies for therapeutic development in nicotine addiction: Rethinking the conventional bench to bedside approach. Prog Neuro-Psychopharmacol Biol Psychiatry.

[24] Field M, Kersbergen I (2020). Are animal models of addiction useful?. Addiction.

[25] Rose JE, Corrigall WA (1997). Nicotine self-administration in animals and humans: similarities and differences. Psychopharmacology (Berl).

[26] Donny EC, Chaudhri N, Caggiula AR, Evans-Martin FF, Booth S, Gharib MA (2003). Operant responding for a visual reinforcer in rats is enhanced by noncontingent nicotine: implications for nicotine self-administration and reinforcement. Psychopharmacology (Berl).

[27] Deroche-Gamonet V, Piat F, Le Moal M, Piazza PV (2002). Influence of cue-conditioning on acquisition, maintenance and relapse of cocaine intravenous self-administration. Eur J Neurosci.

[28] Garcia-Rivas V, Fiancette J-F, Cannella N, Carbo-Gas M, Renault P, Tostain J (2019). Varenicline targets the reinforcing-enhancing effect of nicotine on its associated salient cue during nicotine self-administration in the rat. Front Behav Neurosci.

[29] Olsen CM, Winder DG (2009). Operant sensation seeking engages similar neural substrates to operant drug seeking in C57 mice. Neuropsychopharmacol.

[30] Contet C, Whisler KN, Jarrell H, Kenny PJ, Markou A (2010). Patterns of responding differentiate intravenous nicotine self-administration from responding for a visual stimulus in C57BL/6J mice. Psychopharmacology.

[31] Faul F, Erdfelder E, Lang A-G, Buchner AG. *Power 3: a flexible statistical power analysis program for the social, behavioral, and biomedical sciences. Behav Res Methods. 2007;39:175–91. 10.3758/bf03193146 17695343

[32] Donny EC, Caggiula AR, Knopf S, Brown C (1995). Nicotine self-administration in rats. Psychopharmacology.

[33] Pushparaj A, Pryslawsky Y, Forget B, Yan Y, Foll BL (2012). Extinction bursts in rats trained to self-administer nicotine or food in 1-h daily sessions. Am J Transl Res.

[34] Olfson E, Bloom J, Bertelsen S, Budde JP, Breslau N, Brooks A, et al. CYP2A6 metabolism in the development of smoking behaviors in young adults. Addict Biol. 2018;23:437–47. 10.1111/adb.12477 PMC5491369 28032407

[35] Grebenstein PE, Burroughs D, Roiko SA, Pentel PR, LeSage MG (2015). Predictors of the nicotine reinforcement threshold, compensation, and elasticity of demand in a rodent model of nicotine reduction policy. Drug Alcohol Depend.

[36] Formann AK. Die Latent-Class-Analyse: Einführung in Theorie und Anwendung. Weinheim [W. Germany]: Beltz; 1984.

[37] Cohen C, Perrault G, Griebel G, Soubrié P (2005). Nicotine-associated cues maintain nicotine-seeking behavior in rats several weeks after nicotine withdrawal: reversal by the cannabinoid (CB1) receptor antagonist, rimonabant (SR141716). Neuropsychopharmacology.

[38] Constantin A, Clarke PBS. Reinforcement enhancement by nicotine in adult rats: behavioral selectivity and relation to mode of delivery and blood nicotine levels. Psychopharmacology. 2018;235:641–50. 10.1007/s00213-017-4778-3 29128873

[39] Cohen A, George O (2013). Animal models of nicotine exposure: relevance to second-hand smoking, electronic cigarette use, and compulsive smoking. Front Psychiatry.

[40] Garcia-Rivas V, Cannella N, Deroche-Gamonet V (2017). Individual variations in the mechanisms of nicotine seeking: a key for research on nicotine dependence. Neuropsychopharmacology.

[41] Newby PK, Tucker KL (2004). Empirically derived eating patterns using factor or cluster analysis: a review. Nutr Rev.

[42] Clemens KJ, Lay BPP, Holmes NM (2017). Extended nicotine self-administration increases sensitivity to nicotine, motivation to seek nicotine and the reinforcing properties of nicotine-paired cues. Addict Biol.

[43] Grimm JW, Ratliff C, North K, Barnes J, Collins S (2012). Nicotine increases sucrose self-administration and seeking in rats. Addict Biol.

[44] Rupprecht LE, Smith TT, Schassburger RL, Buffalari DM, Sved AF, Donny EC (2015). Behavioral mechanisms underlying nicotine reinforcement. Curr Top Behav Neurosci.

[45] Perkins KA, Karelitz JL, Boldry MC (2017). Nicotine acutely enhances reinforcement from non-drug rewards in humans. Front Psychiatry.

[46] Palmatier MI, Matteson GL, Black JJ, Liu X, Caggiula AR, Craven L (2007). The reinforcement enhancing effects of nicotine depend on the incentive value of non-drug reinforcers and increase with repeated drug injections. Drug Alcohol Depend.

[47] Levin ME, Weaver MT, Palmatier MI, Caggiula AR, Sved AF, Donny EC (2012). Varenicline dose dependently enhances responding for nonpharmacological reinforcers and attenuates the reinforcement-enhancing effects of nicotine. Nicotine Tob Res.

[48] Perkins KA, Karelitz JL, Michael VC (2015). Reinforcement enhancing effects of acute nicotine via electronic cigarettes. Drug Alcohol Depend.

[49] Liu X, Palmatier MI, Caggiula AR, Donny EC, Sved AF (2007). Reinforcement enhancing effect of nicotine and its attenuation by nicotinic antagonists in rats. Psychopharmacology (Berl).

[50] Perkins KA, Karelitz JL (2013). Influence of reinforcer magnitude and nicotine amount on smoking’s acute reinforcement enhancing effects. Drug Alcohol Depend.

[51] Perkins KA, Karelitz JL (2014). Sensory reinforcement-enhancing effects of nicotine via smoking. Exp Clin Psychopharmacol.

[52] Martin LM, Sayette MA (2018). A review of the effects of nicotine on social functioning. Exp Clin Psychopharmacol.

[53] Perkins KA, Karelitz JL, Boldry MC (2019). Reinforcement enhancing effects of nicotine via patch and nasal spray. Nicotine Tob Res.

[54] Satanove DJ, Rahman S, Chan TMV, Ren S, Clarke PBS (2021). Nicotine-induced enhancement of a sensory reinforcer in adult rats: antagonist pretreatment effects. Psychopharmacology (Berl).

[55] Pergadia ML, Der-Avakian A, D’Souza MS, Madden PAF, Heath AC, Shiffman S (2014). Association between nicotine withdrawal and reward responsiveness in humans and rats. JAMA Psychiatry.

[56] Cook JW, Piper ME, Leventhal AM, Schlam TR, Fiore MC, Baker TB (2015). Anhedonia as a component of the tobacco withdrawal syndrome. J Abnorm Psychol.

[57] Piper ME (2015). Withdrawal: expanding a key addiction construct. Nicotine Tob Res.

[58] Piper ME, Vasilenko SA, Cook JW, Lanza ST (2017). What a difference a day makes: differences in initial abstinence response during a smoking cessation attempt. Addiction.

[59] Perkins KA (2009). Acute responses to nicotine and smoking: implications for prevention and treatment of smoking in lower SES women. Drug Alcohol Depend.

[60] Audrain-McGovern J, Wileyto EP, Ashare R, Cuevas J, Strasser AA (2014). Reward and affective regulation in depression-prone smokers. Biol Psychiatry.

[61] Leventhal AM (2016). The sociopharmacology of tobacco addiction: implications for understanding health disparities. Nicotine Tob Res.

[62] Lee JO, Cho J, Yoon Y, Bello MS, Khoddam R, Leventhal AM (2018). Developmental pathways from parental socioeconomic status to adolescent substance use: alternative and complementary reinforcement. J Youth Adolesc.

[63] Johnson MW, Bickel WK, Kirshenbaum AP (2004). Substitutes for tobacco smoking: a behavioral economic analysis of nicotine gum, denicotinized cigarettes, and nicotine-containing cigarettes. Drug Alcohol Depend.

[64] Donny EC, Jones M (2009). Prolonged exposure to denicotinized cigarettes with or without transdermal nicotine. Drug Alcohol Depend.

[65] Shram MJ, Funk D, Li Z, Lê AD (2008). Nicotine self-administration, extinction responding and reinstatement in adolescent and adult male rats: evidence against a biological vulnerability to nicotine addiction during adolescence. Neuropsychopharmacol.

[66] Tiffany ST, Hakenewerth DM (1991). The production of smoking urges through an imagery manipulation: psychophysiological and verbal manifestations. Addict Behav.

[67] Cepeda-Benito A, Tiffany ST (1996). The use of a dual-task procedure for the assessment of cognitive effort associated with cigarette craving. Psychopharmacology (Berl).

[68] Tiffany ST, Cox LS, Elash CA (2000). Effects of transdermal nicotine patches on abstinence-induced and cue-elicited craving in cigarette smokers. J Consult Clin Psychol.

[69] Dallery J, Houtsmuller EJ, Pickworth WB, Stitzer ML (2003). Effects of cigarette nicotine content and smoking pace on subsequent craving and smoking. Psychopharmacology (Berl).

[70] Barrett SP (2010). The effects of nicotine, denicotinized tobacco, and nicotine-containing tobacco on cigarette craving, withdrawal, and self-administration in male and female smokers. Behav Pharm.

[71] Baker TB, Japuntich SJ, Hogle JM, McCarthy DE, Curtin JJ (2006). Pharmacologic and behavioral withdrawal from addictive drugs. Curr Dir Psychol Sci.

[72] Rose JE, Herskovic JE, Trilling Y, Jarvik ME (1985). Transdermal nicotine reduces cigarette craving and nicotine preference. Clin Pharm Ther.

[73] Brauer LH, Behm FM, Lane JD, Westman EC, Perkins C, Rose JE (2001). Individual differences in smoking reward from de-nicotinized cigarettes. Nicotine Tob Res.

[74] Niaura R, Abrams DB, Pedraza M, Monti PM, Rohsenow DJ (1992). Smokers’ reactions to interpersonal interaction and presentation of smoking cues. Addict Behav.

[75] Conklin CA, Tiffany ST (2001). The impact of imagining personalized versus standardized urge scenarios on cigarette craving and autonomic reactivity. Exp Clin Psychopharmacol.

[76] Van Gucht D, Van den Bergh O, Beckers T, Vansteenwegen D (2010). Smoking behavior in context: where and when do people smoke?. J Behav Ther Exp Psychiatry.

[77] Coe JW, Brooks PR, Vetelino MG, Wirtz MC, Arnold EP, Huang J (2005). Varenicline: an alpha4beta2 nicotinic receptor partial agonist for smoking cessation. J Med Chem.

[78] Rollema H, Coe JW, Chambers LK, Hurst RS, Stahl SM, Williams KE (2007). Rationale, pharmacology and clinical efficacy of partial agonists of alpha4beta2 nACh receptors for smoking cessation. Trends Pharm Sci.

[79] Rollema H, Chambers LK, Coe JW, Glowa J, Hurst RS, Lebel LA (2007). Pharmacological profile of the alpha4beta2 nicotinic acetylcholine receptor partial agonist varenicline, an effective smoking cessation aid. Neuropharmacology.

[80] Ebbert JO, Wyatt KD, Hays JT, Klee EW, Hurt RD (2010). Varenicline for smoking cessation: efficacy, safety, and treatment recommendations. Patient Prefer Adherence.

[81] Falco AM, Bevins RA (2015). Individual differences in the behavioral effects of nicotine: a review of the preclinical animal literature. Pharm Biochem Behav.

[82] Yager LM, Robinson TE (2015). Individual variation in the motivational properties of a nicotine cue: sign-trackers vs. goal-trackers. Psychopharmacology (Berl).

[83] Allenby CE, Boylan KA, Lerman C, Falcone M. Precision medicine for tobacco dependence: development and validation of the nicotine metabolite ratio. J Neuroimmune Pharmacol. 2016;11:471–83. 10.1007/s11481-016-9656-y PMC5479354 26872457

[84] Kazan T, Charntikov S (2019). Individual differences in responding to bupropion or varenicline in a preclinical model of nicotine self-administration vary according to individual demand for nicotine. Neuropharmacology.

[85] Sved AF, Caggiula AR, Donny EC (2023). Elucidating the reinforcing effects of nicotine: a tribute to Nadia Chaudhri. Psychopharmacology (Berl).

[86] Chenoweth MJ, Tyndale RF (2017). Pharmacogenetic optimization of smoking cessation treatment. Trends Pharmacol Sci.

[87] Chen L-S, Horton A, Bierut L (2018). Pathways to precision medicine in smoking cessation treatments. Neurosci Lett.

[88] Siegel SD, Tindle HA, Bergen AW, Tyndale RF, Schnoll R (2023). The use of biomarkers to guide precision treatment for tobacco use. Addict Neurosci.

[89] Bierut LJ (2020). 2018 Langley award for basic research on nicotine and tobacco: bringing precision medicine to smoking cessation. Nicotine Tob Res.

[90] Chen L-S, Baker TB, Ramsey A, Amos CI, Bierut LJ (2023). Genomic medicine to reduce tobacco and related disorders: translation to precision prevention and treatment. Addict Neurosci.

[91] Ahrens S, Markett S, Breckel TPK, Behler O, Reuter M, Thiel CM (2015). Modulation of nicotine effects on selective attention by DRD2 and CHRNA4 gene polymorphisms. Psychopharmacology (Berl).

[92] Bauman KE, Foshee VA, Haley NJ (1992). The interaction of sociological and biological factors in adolescent cigarette smoking. Addict Behav.

[93] Jamner LD, Whalen CK, Loughlin SE, Mermelstein R, Audrain-McGovern J, Krishnan-Sarin S (2003). Tobacco use across the formative years: a road map to developmental vulnerabilities. Nicotine Tob Res.

[94] Cummings KM, Fong GT, Borland R (2009). Environmental influences on tobacco use: evidence from societal and community influences on tobacco use and dependence. Annu Rev Clin Psychol.

[95] Verplaetse TL, Morris ED, McKee SA, Cosgrove KP (2018). Sex differences in the nicotinic acetylcholine and dopamine receptor systems underlying tobacco smoking addiction. Curr Opin Behav Sci.

