# Supplementary material for: Individual variations in motives for nicotine self-administration in male rats: evidence in support for a precision psychopharmacology
Source: Transl Psychiatry. 2024 Feb 9;14:85. doi: 10.1038/s41398-024-02774-6 (PMC10858238; doi:10.1038/s41398-024-02774-6)
Supplement: Supplementary file 1 — Supplementary Material [file 41398_2024_2774_MOESM1_ESM.docx]

**Supplementary Information (SI)**

**Individual variations in motives for nicotine self-administration in male rats: evidence in support for a precision psychopharmacology**

Vernon Garcia-Rivas et al.

**Progressive ratio**

To assess the strength of the reinforcing effects of nicotine in the absence of the associated cue, rats performed two consecutive sessions of progressive ratio. These sessions were identical to the “CueOm” session, except that the ratio of responses per nicotine infusion was increased after each infusion according to the following progression: 3, 6, 10, 15, 20, 25, 32, 40, 50, 62, 77, 95, 118, 145, 179, 219 and 268. The maximum number of responses that a rat performed to obtain one infusion (the last ratio completed) is referred to as the breakpoint. The session ceased after either 3 hours or when 17 infusions were reached (the maximum possible number of infusions).

**Quantification of plasma nicotine and metabolites**

Nicotine (NIC) together with its main metabolites, cotinine (COT) and 3 hydroxy cotinine (OHCOT), were measured in rat plasma samples using a liquid chromatography with tandem mass spectrometry detection (LC-MS/MS) method. Briefly, 100 µL of each plasma sample were mixed with 300 µL of internal standards (NIC-D_4_, COT-D_3_ and OHCOT-D_3_) mixture in methanol. After centrifugation, 100 µL of 5 mM ammonium formate buffer at pH 3 were added to 100 µL of the obtained supernatant: 10 µL were injected in the chromatographic system. Chromatographic separation was performed using an Acquity™ UPLC HSS C18 (1.8 µm, 2.1×150 mm, Waters) column and a gradient of 5 mM ammonium formate / 0.1 % formic acid buffer, and ACN / 0.1 % formic acid as mobile phase. Xevo TQ-S tandem mass spectrometer (Waters) was used for detection after positive electrospray ionization mode in the MRM mode (ESI+) using the following transitions: m/z 163.2 → 132 and 130 (NIC), m/z 167.1 → 136 (NIC-D_4_), m/z 177.1 → 79.9 and 98 (COT), m/z 180.1 → 100.9 (COT-D_3_), m/z 193.1 → 80 and 134 (OHCOT) and m/z 196.1 → 80 (OHCOT-D_3_). In compliance with both the French Analytical Toxicology Society (SFTA) and international recommendations for the validation of new analytical methods [1,2], an additional validation step was performed, which included six independent calibrations conducted on different days and using different rat plasma-free samples. Linearity was determined using linear regression with 1/x weighting for the 3 compounds. The limit of detection (LOD) was defined as the lowest concentration with retention time within ± 0.2 min from the average of all calibrator concentrations and a signal-to-noise ratio of at least three for all selected ion transitions. The lower limit of quantification (LLOQ) was the lowest concentration that could be quantified with acceptable imprecision (CV % ≤ 20 %) and acceptable accuracy (within ± 20 % of the theoretical concentration). Within-day and between-day precision and accuracy were calculated from six repeated analyses of spiked rat plasma samples (at three levels) during one working day, for 6 days.

Immediately after the end of standard session 21, 400 µL blood were collected from the catheter for the quantification of plasma nicotine and metabolites. Blood was put in heparin-containing microtubes (Sarsted 41.1393.005), mixed and placed immediately on ice. Samples were kept on ice until centrifuged (760 G, 10 min, 4°C). Once plasma was separated, 100 µL were carefully pipetted out, placed in 500 µL Eppendorf tubes and stored at -80°C.

**Data analyses**

Variables of interest in FR self-administration sessions were active and inactive responses, reinforcers earned. Loading Proportion in standard FR3 sessions and breakpoint in PR sessions were also considered. As described in the main text, effects of cue and nicotine omission on nicotine self-administration were evaluated through the *Omission Global Effect (Om-GE)* and the *Omission Loading Effect (Om-LE)*. In the disconnection test mean intervals between cues and infusions and between infusions and next cues were calculated over sessions 2 to 4 (sessions 36 to 38).

**Supplementary Results**

***Quantification of Plasma nicotine and metabolites***

LOD and LLOQ for NIC, COT and OHCOT in plasma samples were 0.5 µg/L. Linear regression with 1/x weighting showed the standard curves (n=6) to be linear from 0.5 to 100 µg/L, with r > 0.999, and observed inter-day CV and bias (n=6) were less than 20%.

Consistent with the literature [4], and different from humans where OHCOT and COT concentrations are in a close range, OHCOT levels were low (15.25 ± 1.2 ng/mL) compared to cotinine (328.7 ± 37 ng/mL) and nicotine levels (3791 ± 564 ng/mL). Hence, we used the *main metabolite (COT) / parent drug (NIC) ratio* as an index for metabolism. We found a sustained correlation between this ratio and the total infusions earned (**FigS2c**), while nicotine levels were unrelated to total infusions earned [r=-0.46, r^2^=0.21, p=0.13]. This supports that variations in nicotine metabolism impact nicotine intake in our basal self-administration procedure in accordance with previous studies [5]. Our two clusters did not differ for basal nicotine self-administration behaviour; this argues for no difference in nicotine metabolism between the two clusters.

***Acquisition of self-administration***

*Nicotine+cue self-administration*

Rats acquired and stabilized *nicotine+cue* self-administration behavior during the initial 12 sessions. Mean total self-infusions per session increased [Session effect, F(11,671)=68.18, p<0.0001] to start stabilizing around 30 infusions per session from session 5 (**FigS2a**). Mean total active nose-pokes per session were significantly higher than mean total inactive nose-pokes [Hole effect, F(1,61)=1222, p<0.0001] and the difference amplified over sessions [Hole x Session, F(11,671)=58.23, p<0.0001] (**FigS2b**).

*Saline+cue self-administration*

As previously shown [3], rats self-administered *saline+cue,* as attested by the higher number of active than inactive nose-pokes per session [Hole effect, F(1,7)=35.13, p<0.001] (**FigS2f**)*.* The difference between active and inactive hole visits remained constant over sessions in the *saline+cue* group [Hole x Session, F(11,77)=1.01, p=0.44], as did the number of infusions per session [Session, F(11,77)=1.105, p=0.37] (**FigS2e**). Compared to session 12, cue omission during session 13 resulted in a significant decrease in self-infusions in the *saline+cue* group [Session effect, F(1,7)=34.46, p<0.001]. This effect was reversed by standard *saline+cue* conditions on session 14, providing further support for the hypothesis that the cue was serving as a reinforcer (see **FigS2f**).

*Comparison of nicotine+cue and saline+cue self-administration*

When compared to the *nicotine+cue* group (**FigS2-a&e**), the mean number of infusions per session in the *saline+cue* was significantly lower [Group effect, F(1,68)=14.23, p<0.0005]. The progression over sessions of the mean number of infusions per session was also different [Group x Session, F(11,748)=7.69, p<0.0001].

Motivation for nicotine, as measured through progressive ratio schedule during CueOm (sessions 26-27), was lower in *saline+cue* rats (**FigS2h**) compared to *nicotine+cue* rats (**FigS2d**) [Group effect, F(1,66)=15.03, p<0.0005]. This group difference was sustained over the two progressive ratio sessions [Group x session, F(1,66)=3.006, p=0.09].

Tested on session 23, in parallel to the test in the *nicotine+cue* rats, Varenicline increased self-administration behaviour in *saline+cue* rats (**FigS7**), consistent with a partial agonist nicotine-like enhancing effect. The difference was observed either comparing the *saline+cue* group to the two clusters [Group effect, F(2,60)=7.92, p<0.001] (**FigS7**) or comparing the *saline+cue* group to the whole *nicotine+cue* group [Group effect, F(1,61)=7.1, p<0.01].

***Differences between clusters in CueOm and NicOm effects***

CueOm resulted in a decrease in total infusions [CueOm effect, F(1,60)=318, p<0.00001], with a smaller decrease observed in Cluster B compared to Cluster A [Cluster effect, F(1,60)=23.64, p<0.00001] (**FigS4a**). Additionally, CueOm led to an increase in the loading proportion, but the increase was smaller in Cluster B compared to Cluster A [Cluster effect, F(1,60)=13.58, p<0.0005] (**FigS4b**).

NicOm had a different effect on the two clusters. It decreased total infusions in Cluster A and increased them in Cluster B [Cluster effect, F(1,60)=44.53, p<0.00001] (**FigS4c**). Additionally, NicOm increased the loading proportion in both clusters, but the increase was more pronounced in Cluster A compared to Cluster B [Cluster effect, F(1,60)=13.11, p<0.001] (**FigS4d**).

**Supplementary references**

1. Peters FT, Drummer OH, Musshoff F. Validation of new methods. Forensic Sci Int. 2007;165:216–224.

2. Wille SMR, Peters FT, Fazio V, Samyn N. Practical aspects concerning validation and quality control for forensic and clinical bioanalytical quantitative methods. Accreditation and Quality Assurance. 2011;6:279–292.

3. Garcia-Rivas V, Fiancette J-F, Cannella N, Carbo-Gas M, Renault P, Tostain J, et al. Varenicline Targets the Reinforcing-Enhancing Effect of Nicotine on Its Associated Salient Cue During Nicotine Self-administration in the Rat. Front Behav Neurosci. 2019;13:159.

4. Craig EL, Zhao B, Cui JZ, Novalen M, Miksys S, Tyndale RF. Nicotine pharmacokinetics in rats is altered as a function of age, impacting the interpretation of animal model data. Drug Metab Dispos. 2014;42:1447–1455.

5. Grebenstein PE, Burroughs D, Roiko SA, Pentel PR, LeSage MG. Predictors of the nicotine reinforcement threshold, compensation, and elasticity of demand in a rodent model of nicotine reduction policy. Drug Alcohol Depend. 2015;151:181–193.

**Supplementary Table 1**

**Supplementary Table 2**

**Supplementary Table 3**

**Supplementary Figure 1**

**FigS1: Time course of infusions per 30 min time bins in rats self-administering nicotine+cue.** Mean number of infusions (± SEM) and individual values per 30 min time bins over the 3hrs of basal self-administration sessions 11 and 12. After an initial loading phase, intake becomes fully regular in mean starting bin T60-T90. Based on this profile, T30-60 still being different from T90-T120, we chose time 60 min as the end of the loading phase.

**Supplementary Figure 2**


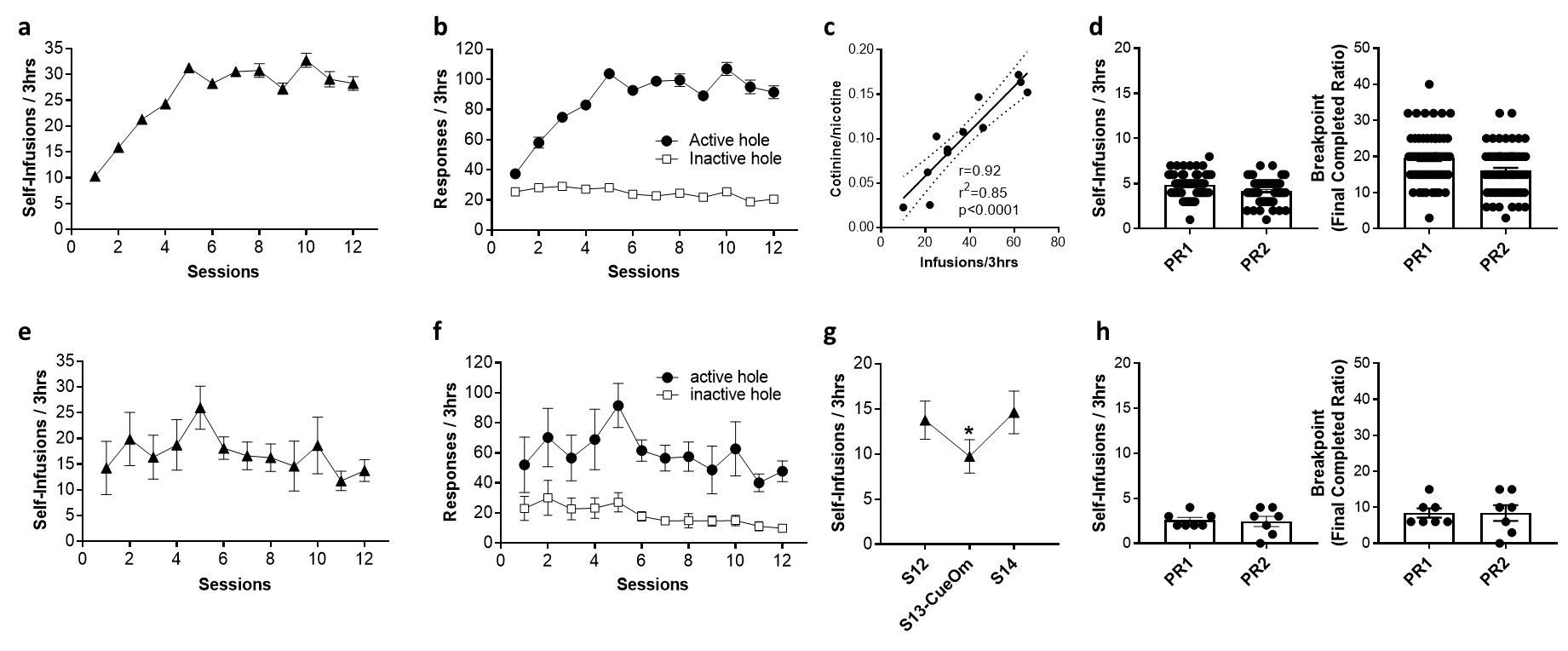


**FigS2: Acquisition of intravenous self-administration behaviour in i.v. nicotine+cue and i.v. saline+cue rats and relation of nicotine self-administration with cotinine/nicotine ratio. a.** Mean self-infusions earned per session by the nicotine+cue group. **b.** Mean active and inactive responses per session by the nicotine+cue group. **c.** Correlation between nicotine infusions earned over a 3hr-session in nicotine+cue rats and plasma cotinine/nicotine ratio. **d.** Self-infusions and breakpoint over the two progressive ratio (PR) sessions in the nicotine+cue group. **e.** Mean self-infusions earned per session by the saline+cue group. **f.** Mean active and inactive responses per session by the saline+cue group. **g.** Effect of cue omission (CueOm) on session 13 in the saline+cue group. *p<0.05 as compared to session 12 (S12) and session 14 (S14). **h.** Self-infusions and breakpoint over the two progressive ratio (PR) sessions in the saline+cue group. **a-b;d-h:** Data are expressed as mean±sem.

**Supplementary Figure 3**

**
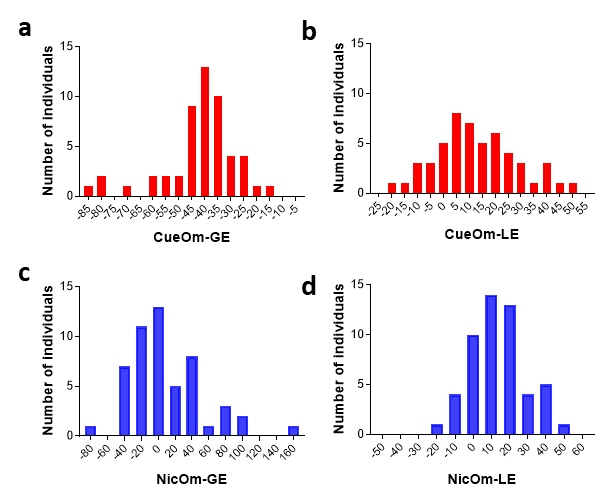
**

**FigS3: Distributions of Individual Scores in Global Effect (GE) and Loading Effect (LE) for CueOmission (CuOm) and Nicotine Omission (NicOm) Tests.** (**a**) Distribution of individual scores in GE for CuOm. (**b**) Distribution of individual scores in LE for CuOm. (**c**) Distribution of individual scores in GE for NicOm. (**d**) Distribution of individual scores in LE for NicOm.

**Supplementary Figure 4**

**
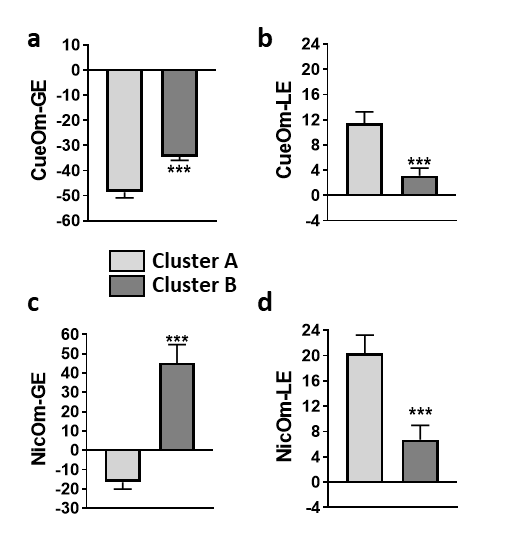
**

**FigS4:** **Differences between Clusters A and B in Global Effect (GE) and Loading Effect (LE) of Cue Omission (a-b) and Nicotine Omission (c-d).** Data are presented as mean ± SEM. ***p < 0.001.

**Supplementary Figure 5**

**FigS5: First 20 min of the disconnection test.** Cumulative time course of active and inactive responses in the two holes for clusters A and B. Over the first 20 min, visits to the cue hole have no consequences, while visits to the nicotine hole are reinforced by nicotine infusions. Data are expressed as mean±sem.

**Supplementary Figure 6**

**FigS6: First session of the disconnection test.** Same as FigS5 but over the entire sessions (3hrs). After 20 min, the two holes are reinforced. Data are expressed as mean±sem.

**Supplementary Figure 7**

**
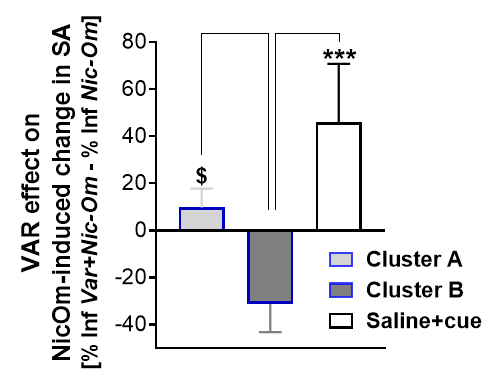
**

**FigS7: Varenicline effect on nicotine omission test. Same as Fig4i-right but including the *saline+cue* group.** $p<0.05, ***p<0.001. Data are expressed as mean±sem.
